# Co-administration of GnRH-agonist and hCG for final oocyte maturation (double trigger) in patients with low number of oocytes retrieved per number of preovulatory follicles-a preliminary report

**DOI:** 10.1186/1757-2215-7-77

**Published:** 2014-08-02

**Authors:** Jigal Haas, Eran Zilberberg, Shir Dar, Alon Kedem, Ronit Machtinger, Raoul Orvieto

**Affiliations:** 1 Department of Obstetrics and Gynecology, Chaim Sheba Medical Center (Tel Hashomer), Ramat Gan, Israel; 2 Sackler Faculty of Medicine, Tel Aviv University, Tel Aviv, Israel

**Keywords:** Oocytes retrieval, GnRH-antagonist, hCG, GnRH-agonist, Final oocyte maturation, IVF outcome

## Abstract

**Background:**

Recently, the co-administration of GnRH agonist and hCG for final oocyte maturation- 40 and 34 hours prior to OPU, respectively (double trigger) was suggested as the treatment of genuine empty follicle syndrome. In the present study, we aim to evaluate whether the double trigger improves the number of oocytes retrieved in patients with low (<50%) number of oocytes retrieved per number of preovulatory follicles.

**Methods:**

In this proof of concept cohort historical study, we compared the stimulation characteristics of 8 IVF cycles, which include the double trigger to the patients’ previous IVF attempt, triggered with hCG-only.

**Results:**

Patients who received the double trigger (study group) had a significantly higher number of oocytes retrieved, number of 2PN, number of embryos transferred and significantly higher proportions of the number of oocytes retrieved to the number of follicles >10 mm and >14 mm in diameter on day of hCG administration, with a tendency toward a higher number of TQE, as compared to their previous cycles (hCG-only trigger). Three ongoing clinical pregnancies were recorded in the study group and none in the hCG-only trigger group.

**Conclusions:**

Co-administration of GnRH-agonist and hCG for final oocyte maturation, 40 and 34 hours prior to OPU, respectively (double trigger), is suggested as a valuable new tool in the armamentarium for treating patients with low/poor oocytes yield despite an apparently normal follicular development and E2 levels and in the presence of optimal hCG levels on the day of OPU.

## Background

Controlled ovarian hyperstimulation (COH) is considered a key factor in the success of in vitro fertilization-embryo transfer (IVF-ET) because it enables the recruitment of multiple healthy fertilizable oocytes [1]. Moreover, human chorionic gonadotropin (hCG) is usually used at the end of COH, as a surrogate LH surge, to induce final oocyte maturation and resumption of meiosis [2].

Lack of oocyte yield during ovum pick-up (OPU), following COH with an apparently normal follicular development and E2 levels and in the presence of optimal hCG levels on the day of OPU- the genuine empty follicle syndrome (EFS) [3], is a rare entity, with an estimated prevalence of 0–1.1% [4,5]. On the other hand, a more frequently encountered situation is the normally responding patients with a low oocytes yield, i.e. a low ratio (<50%) between the number of oocytes retrieved to the number of follicles >14 mm in diameter on the day of hCG administration.

Despite many years of clinical experience [6], the underlying mechanism of the aforementioned conditions is still obscure and no precise prevention methods exist [5]. Several strategies were offered to patients with EFS [summarized in [5]], including another standard ART cycle; shifting from an GnRH-agonist to GnRH-antagonist COH protocol; re-administering hCG from a different batch and aspirating the second ovary; changing the hCG from a urinary to a recombinant preparation; using GnRH-agonist for final; prolonging the interval between ovulation triggering and OPU; and follicle flushing during oocyte retrieval [7].

Recently, a new treatment modality has been clinically implemented to EFS patient, with the co-administration of GnRH agonist and hCG for final oocyte maturation- 40 and 34 hours prior to OPU, respectively (double trigger) [5]. This method combines the advantage of both: (1) the prolongation of the time between ovulation triggering and OPU; and (2) the GnRH agonist trigger with the consequent simultaneous induction of an FSH surge.

Prompted by this new remedy, we offered the double trigger to all our patients, who underwent the GnRH-antagonist COH protocol, resulting with poor oocytes yield due to low (<50%) number of oocytes retrieved per number of follicles > 14 mm in diameter on day of hCG administration. In the present study, we aim to further evaluate whether the double trigger improves the number of oocytes retrieved and the ratio between the number of oocytes retrieved per number of follicles > 14 mm in diameter on day of hCG administration.

## Methods

All consecutive patients with poor oocytes yield, despite normal response to COH, due to low (<50%) number of oocytes retrieved per number of follicles > 14 mm in diameter on day of hCG administration, who were treated in our IVF unit during one year period were evaluated. Of whom, only those who received in the subsequent IVF cycle, a double trigger (GnRH-agonist and hCG) for final follicular maturation were included. The study was approved by the Institutional Research Ethics Board of our center.

All patients underwent the multi-dose GnRH-antagonist COH protocol during both IVF cycles. In both cycles, ovulation induction was performed by the administration of recombinant FSH, started at the 2nd or 3rd day of menses, using the same starting dose in each patient. Once the leading follicle had reached a size of 13 mm, or/and E2 levels exceeded 1200 pmol/L, co-treatment with the GnRH antagonist 0.25 mg/day, was initiated and the recombinant FSH was substituted by human menopausal gonadotropins. Gonadotropins doses were further adjusted according to serum estradiol levels and vaginal ultrasound measurements of follicular diameter, obtained every two or three days. Final follicular maturation was triggered by, either:

(1) In the first IVF cycles: recombinant hCG (Choriogonadotropin alfa, ovitrelle 250 mcg, Serono), 36 hours prior to OPU, or; (2) In subsequent cycles: the co-administration of GnRH-agonist (Triptorelin acetate, decapeptyl 0.2 mg, Ferring Pharmaceuticals, Israel) and recombinant hCG (250 mcg), 40 and 34 hours prior to oocyte retrieval, respectively.

Routine IVF or intracytoplasmic sperm injection (ICSI) was then performed, as appropriate. Transvaginal ET was performed 48 to 72 hours after OPU in both cycles. All patients received luteal support with progesterone.

Data on patient age and infertility-treatment-related variables were collected from the files. Ovarian stimulation characteristics, number of follicles >10 mm and >14 mm in diameter on day of hCG administration, number of oocytes retrieved, embryo quality and number of embryos transferred were assessed and compared between the study (double trigger) cycle and the previous (hCG-only trigger) control cycle.

Embryos classification was based on the individual embryo scoring parameters according to pre-established definitions [8]. While a top quality embryo (TQE) was defined as seven or more blastomeres on day 3, equally-sized blastomeres and <20% fragmentation, poor quality embryos consist of all the rest. Clinical pregnancy was defined as visualization of a gestational sac and fetal cardiac activity on transvaginal ultrasound.

Statistical analysis was performed with Student’s paired t-test and chi square, as appropriate. Results are presented as means ± standard deviations; p value < 0.05 was considered significant.

## Results

Eight consecutive patients were evaluated. Mean age and body mass index during the study cycle were 38.0 ± 4.8 years and 27.7 + 5.4 kg/m^2^, respectively. The clinical characteristics of the IVF cycles in the two study cycles are shown in Table 1.

**Table 1:** Comparison between IVF cycles with hCG versus double trigger (GnRH-ag + hCG)

	**hCG**	**Double trigger**	**p values**
Number of gonadotropin ampoules used	38.5 ± 24.5	49.8 ± 15.4	ns
Length of stimulation (days)	10.7 ± 2.5	10.7 ± 2.2	ns
Total number of gonadotropin used	39 ± 25	49 ± 15	ns
Peak E2 levels on day of hCG administration (pmol/l)	5402 ± 1983	4642 ± 2483	ns
Progesterone levels on day of hCG administration (nmol/l)	1.7 ± 0.9	1,5 ± 0.7	ns
Number of follicles of >14 mm on day of hCG administration	8.0 ± 3.5	6.4 ± 4.3	ns
Number of follicles of >10 mm on day of hCG administration	10.6 ± 5.8	8.2 ± 4.3	ns
Number of oocytes retrieved	2.3 ± 2.5	7.0 ± 4.6	p < 0.02
Number of 2PN embryos	1.7 ± 1.2	6.0 ± 4.6	p < 0.002
Number of top quality embryos	0.4 ± 0.5	3.7 ± 0.8	p = 0.06
Number of embryos transferred	0.85 ± 0.9	2.2 ± 0.7	p < 0.002
Number of oocytes retrieved per number follicles of >14 mm on day of hCG administration (%)	23.7 ± 21.5	118.0 ± 71.2	p < 0.01
Number of oocytes retrieved per number follicles of >10 mm on day of hCG administration (%)	18.5 ± 16.6	80.3 ± 31.1	p < <0.001
Positive hCG (%)	0 (0/8)	62.5% (5/8)	p < 0.001
Clinical ongoing pregnancy (%)	0 (0/8)	37.5% (3/8)	p < 0.03

In the present study, while using the same COH protocols which differ only in the methods of triggering final follicular maturation, as expected, no differences were observed between the groups in the length of stimulation, the number of gonadotropin ampoules administered, peak estradiol and progesterone levels and numbers of follicles >10 mm and >14 mm in diameter on day of hCG administration. However, patients who received the double trigger (study group) had a significantly higher number of oocytes retrieved (7.0 ± 4.6 vs. 2.3 ± 2.5, p < 0.02), number of 2PN (6.0 ± 4.6 vs. 1.7 ± 1.2, p < 0.002), number of embryos transferred (2.2 ± 0.7 vs. 0.85 ± 0.9, p < 0.002) and significantly higher proportions of the number of oocytes retrieved to the number of follicles >10 mm (80.3% ±31.1% vs. 18.5% ± 16.6%, p < 0.001) and >14 mm in diameter (118.0% ± 71.2% vs. 23.7% ± 21.5%, p < 0.01) on day of hCG administration, with a tendency toward a higher number of TQE (3.7 ± 0.8 vs. 0.4 ± 0.5, p = 0.06) respectively, as compared to the hCG-only trigger cycles.

Five pregnancies were recorded in the study group and none in the hCG-only trigger group (Table 1). Of which, 3 are ongoing, one resulted in a blighted ovum and one was biochemical pregnancy.

## Discussion

In the present preliminary cohort historical study, the co-administration of GnRH agonist and hCG for final oocyte maturation- 40 and 34 hours prior to OPU, respectively, to patients with poor oocytes yield due to low (<50%) number of oocytes retrieved per number of follicles > 14 mm in diameter on day of hCG administration, resulted in significantly higher numbers of oocytes’ retrieved and embryos transferred and a significantly higher proportion of the number of oocytes’ retrieved to number of preovulatory follicles. Of notice, the observed improved in oocyte yield was despite a non-significant decrease in the number of follicles of >14 mm and >10 mm on day of hCG administration.

Five biochemical (positive hCG) pregnancies were recorded in the study group (double trigger) and none in the hCG-only trigger group. Of which, 3 (37.5%) are ongoing and one resulted in a blighted ovum. However, it should be emphasized that the increased pregnancy rate in the study group (double trigger) is biased due to the study design which offered this protocol to patients who had failed a previous IVF attempt.

As part of a standard/conventional COH regimen, final oocyte maturation and resumption of meiosis are usually triggered by one bolus of hCG (5000–10,000 units), that is administered as close as possible to the time of ovulation (i.e. 36 hours before oocyte recovery) [2]. In 1990, Gonen et al. [9] have demonstrated that ovulation may be also triggered by GnRH agonist, causing the release of both endogenous LH and FSH, while in 2008, Shapiro et al. [10] have established the concept of ‘Dual trigger’, combining both hCG and GnRH-agonists, aiming to trigger ovulation with the questionable ability to prevent severe ovarian hyperstimulation syndrome [11,12].

Recently, Beck-Fruchter et al. [5] have described a case of recurrent empty follicle syndrome, successfully treated by ovulation trigger with GnRH agonist 40 hours and hCG added 34 hours prior to OPU. They assumed that by prolonging the time between ovulation triggering and OPU and the GnRH agonist trigger with the consequent simultaneous induction of an FSH surge, the “double trigger” could overcome any existing impairments in granulosa cell function, oocyte meiotic maturation or cumulus expansion, resulting in successful aspiration of mature oocytes, pregnancy and delivery.

In the present preliminary report, we further extended the aforementioned indications to the “double trigger” and offered it to patients with poor oocytes yield due to low (<50%) number of oocytes retrieved per number of follicles > 14 mm in diameter on day of hCG administration, despite an apparently normal follicular development and E2 levels during COH. While number of follicles of >14 mm and >10 mm on day of hCG administration wwere non-significantly decrease, we could demonstrate a significant increase in oocytes yield, a trend toward a higher number of TQE, with a reasonable clinical pregnancy rate. These observations are in agreement with Lin et al. [13], who compared the IVF outcome of normal responders, undergoing triggering of final oocyte maturation with either hCG and GnRH-agonist, both administered 35-36 hours prior to OPU, or hCG-only. The double trigger group demonstrated statistically significantly higher implantation, clinical pregnancy and live-birth rates as compared with the hCG trigger group.

## Conclusions

“Double trigger” is suggested as a valuable new tool in the armamentarium for treating patients with low/poor oocytes yield, despite an apparently normal follicular development and E2 levels and in the presence of optimal hCG levels on the day of OPU. Further large prospective studies are needed to elucidate the aforementioned recommendation in this and other situations, such as patients with high proportion of immature oocytes or poor responder patients, prior to its routine implementation.

## Abbreviations

COH: Controlled ovarian hyperstimulation; EFS: Empty follicle syndrome; hCG: Human chorionic gonadotropin; IVF-ET: In vitro fertilization-embryo transfer; OPU: Ovum pick-up; TQE: Top quality embryo.

## Competing interests

The authors declare that they have no competing interests.

## Authors’ contributions

JH- Participated in the clinical management and collected the data, performed the statistical evaluations, assisted in writing the paper and edited it in all its revisions. EZ- Collected the data, performed the statistical evaluations, assisted in writing the paper and edited it in all its revisions. SD- Participated in the clinical management and edited the paper in all its revisions. AK- Participated in the clinical management and edited the paper in all its revisions. RM- Participated in the clinical management and edited the paper in all its revisions. RO- The principal investigator, designed the study, participated in the clinical management, performed the statistical evaluations, assisted in writing the paper and edited it in all its revisions. All authors read and approved the final manuscript

## Authors’ information

Drs. Haas and Zilberberg should be considered “similar in author order.”
